# Mapping Efficiency of an Octaspline Versus Pentaspline Multielectrode Catheter for High-Density Mapping of Regular Atrial Tachyarrhythmias: A Multicenter Randomized Cross-Over Trial

**DOI:** 10.3390/jcdd13080379

**Published:** 2026-08-10

**Authors:** Bakhtawar K. Mahmoodi, Jippe C. Balt, Muchtiar Khan, Maurits C. E. F. Wijffels, Gijsbert S. de Ruiter, Tamas Szili-Torok, Sing-Chien Yap

**Affiliations:** 1 Department of Cardiology, Thorax Center, Cardiovascular Institute, Erasmus Medical Center, 3015 GD Rotterdam, The Netherlands; 2 Department of Cardiology, St. Antonius Hospital, 3435 CM Nieuwegein, The Netherlands; 3 Department of Cardiology, Hospital Onze Lieve Vrouwe Gasthuis, 1091 AC Amsterdam, The Netherlands; 4 Department of Internal Medicine, Cardiology Center, University of Szeged, 6725 Szeged, Hungary

**Keywords:** atrial tachyarrhythmias, catheter ablation, high-density mapping, Octaray catheter, Pentaray catheter, electrogram acquisition

## Abstract

**Background**: Catheter ablation of regular atrial tachyarrhythmias (ATs) relies on high-density electroanatomical mapping and can be technically challenging. We compared the mapping efficiency of the Octaray and Pentaray catheters in patients undergoing AT mapping. **Methods**: Patients with either left or right AT underwent three-dimensional Coherent mapping using both the Octaray and Pentaray catheters in a randomized cross-over design. The primary endpoint was the total number of electrograms acquired per map at a predefined filling threshold of 5 mm. **Results**: A total of 46 patients with left or right AT were enrolled across three ablation centers. Successful mapping with both catheters was achieved in 39 patients (20 left ATs and 19 right ATs). The median number of electrograms acquired per map (7437 vs. 4679; *p* < 0.001) and median electrogram density (50.2 vs. 29.8 electrograms/cm^2^; *p* < 0.001) was significantly higher with Octaray than with Pentaray. Furthermore, median mapping times were significantly shorter with Octaray (5.4 vs. 8.1 min; *p* < 0.001), resulting in an approximately 2.5-fold higher acquisition rate (1489 vs. 602 electrograms/minute; *p* < 0.001). In addition, Octaray demonstrated a significantly lower proportion of internal points compared with Pentaray, particularly in low-voltage regions (bipolar voltage < 0.5 mV; 22% vs. 44%; *p* = 0.01). **Conclusions**: Compared with Pentaray, use of the Octaray catheter facilitated faster high-density electroanatomical mapping of complex ATs, thereby improving mapping efficiency.

## 1. Introduction

The management of atrial fibrillation has evolved substantially over recent decades, with catheter ablation becoming an established rhythm-control strategy [1,2]. As the number of atrial fibrillation ablation procedures continues to increase, the incidence of post-ablation atrial tachyarrhythmias (ATs), particularly atypical atrial flutters, has also risen [3,4,5]. These arrhythmias commonly result from conduction recovery across prior ablation lines or from areas of spontaneous atrial scar that create zones of slow conduction [6,7]. Compared with atrial fibrillation, ATs are often more symptomatic and less responsive to antiarrhythmic drug therapy, making effective mapping and ablation clinically important.

Successful treatment of ATs depends on accurate identification of the underlying mechanism, including focal, micro-reentrant, and macro-reentrant circuits [6,7]. This often requires high-density electroanatomical mapping because arrhythmia circuits may involve complex conduction pathways and extensive atrial scar [7,8,9]. The CARTO 3 mapping system (Johnson & Johnson MedTech, Irvine, CA, USA), including the Coherent Mapping algorithm, integrates local activation timing and anatomical information to facilitate visualization of complex arrhythmia propagation [10].

The quality and interpretability of electroanatomical maps remain highly dependent on mapping catheter design and signal processing characteristics [7]. The recently developed Octaray catheter has more splines (8 vs. 5), more electrodes (48 vs. 20), and smaller electrode surface areas (0.9 vs. 2.0 mm^2^) compared with the Pentaray catheter [11]. Furthermore, the Pentaray catheter uses the Wilson Central Terminal as the reference electrode to record unipolar electrograms, while the Octaray catheter uses a close indifferent electrode embedded in the catheter shaft (TRUEref™ technology, Biosense Webster, Irvine, CA, USA) to reduce far-field potentials and incorrect annotation [12]. Preclinical studies have suggested improved mapping performance of the Octaray catheter compared with the Pentaray catheter [11,13]. However, clinical data evaluating the procedural mapping efficiency of Octaray during mapping of regular ATs remain limited [14,15,16].

Therefore, this randomized cross-over study aimed to compare the mapping efficiency of the Octaray and Pentaray catheters during mapping of left and right ATs.

## 2. Methods

### 2.1. Trial Design

**Figure 1:**
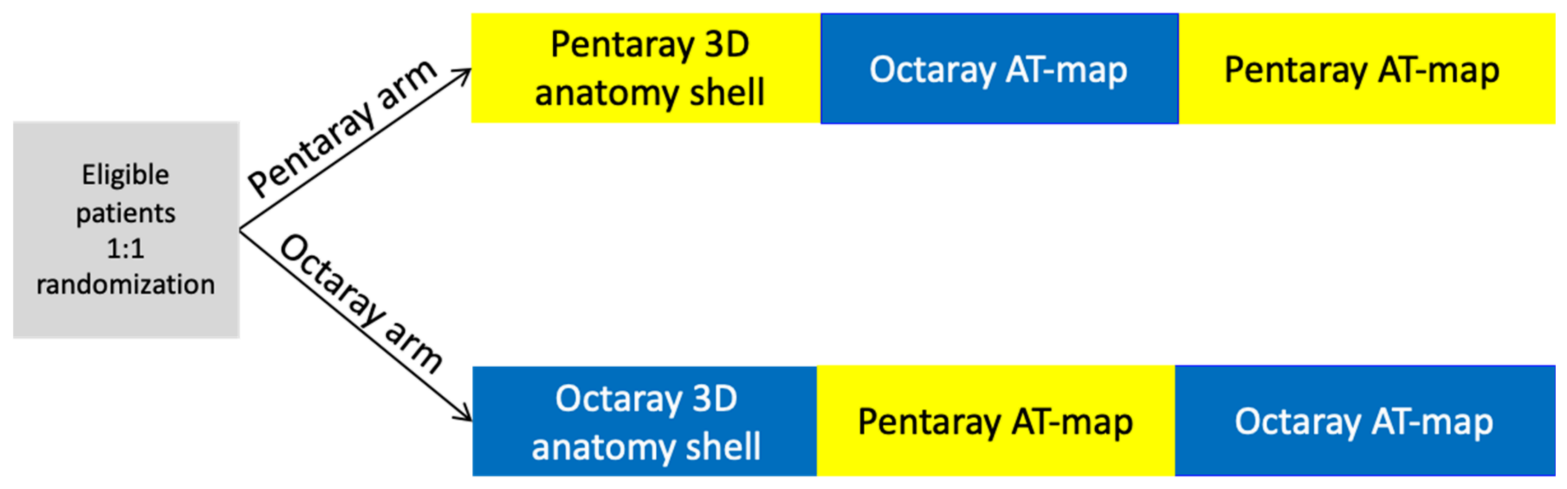
Study design and randomization scheme. Eligible patients were randomized 1:1 to initial 3D anatomy mapping with Pentaray (yellow boxes) or Octaray (blue boxes), followed by alternating atrial tachycardia (AT) mapping with both catheters.

This was a randomized, open-label, cross-over trial including consecutive patients scheduled for ablation of either left or right AT, including typical cavotricuspid isthmus–dependent flutter. The study was designed to compare procedural mapping efficiency between the novel Octaray catheter and the conventional Pentaray multielectrode catheter. The Octaray catheter with a 2-5-2-5-2 mm electrode spacing was selected over the 3-3-3-3-3 and 2-2-2-2-2 configuration because it more closely resembles the 2-6-2 mm spacing of the Pentaray catheter. Eligible patients were approached for participation at least seven days prior to the ablation procedure. The atrial chamber of origin for the AT was determined at the beginning of the procedure by placing a decapolar catheter in the coronary sinus, with or without additional entrainment maneuvers. Following confirmation of sustained AT and identification of the involved chamber, the order of the mapping catheter (Octaray first or Pentaray first) was assigned by randomization (Figure 1). Due to the cross-over study design, every patient was mapped with both the Octaray and Pentaray catheter.

### 2.2. Study Participants

Adult patients (age ≥ 18 years) planned for catheter ablation of either left or right AT, and who were able to provide written informed consent, were eligible for inclusion. Patients with typical cavotricuspid isthmus flutters were also eligible for the right AT group. Patients were recruited and planned for procedures at one of the three participating high-volume ablation centers (i.e., Sint Antonius Hospital in Nieuwegein; Onze Lieve Vrouwe Gasthuis in Amsterdam; and Erasmus Medical Center in Rotterdam) in the Netherlands. At each participating center, study procedures were performed by up to two experienced electrophysiologists familiar with both mapping catheters. The study protocol was approved by the institutional review board of each hospital and every patient provided written informed consent.

### 2.3. Ablation Procedures

All procedures were performed with CARTO 3 (version 7.5 or 8.0) three-dimensional electroanatomical mapping support. Following femoral vein access, a decapolar catheter was positioned in the coronary sinus. In patients presenting in sinus rhythm, AT was induced using programmed extrastimulation and/or burst pacing. Patients in whom AT could not be induced were classified as screen failures.

Once AT was induced, an attempt was made to identify the most likely atrial chamber of origin (i.e., right or left atrium) using entrainment maneuvers, if necessary. Upon confirmation of sustained AT and identification of the likely chamber of origin, patients were randomized. The random allocation sequence was generated using the built-in computer-based randomization module of Castor Electronic Data Capture (Castor EDC). Randomization was performed using variable block randomization with block sizes of 2 and 4 and a 1:1 allocation ratio. Patients were randomized in a cross-over design to either the Octaray-first or Pentaray-first mapping sequence. Randomization was stratified by chamber of origin (left atrium versus right atrium).

For example, as illustrated in Figure 1, if a patient was randomized to the Octaray-first group, an anatomical shell of the chamber of interest was first created using the Octaray catheter, with the local activation timing (LAT) map hidden from the operator during this initial map. Once the complete chamber anatomy was established, key anatomical landmarks such as valves, pulmonary veins, or caval veins were defined. Subsequently, this anatomical shell served as the reference structure for LAT re-mapping with both catheters to ensure harmonization of the total mapped area. The comparator catheter, i.e., the one to which the patient was not randomized, was used first to acquire the LAT re-map. This was followed by LAT re-mapping with the randomized (assigned) catheter. This sequence was selected to minimize bias related to prior chamber acquisition and map completion. A LAT map was considered complete once the entire chamber surface was mapped with an interpolation threshold of <5 mm [11,13]. The high- and low-pass filters settings were harmonized between the catheters as well as other setting such as tissue proximity indication. In addition to LAT maps, simultaneous bipolar voltage maps during AT were acquired; areas with bipolar voltage of <0.5 mV were considered scar tissue. After the LAT maps were acquired, the study phase was concluded. Operators were then free to proceed with the remainder of the procedure according to their standard practice, including the use of either catheter for any additional remapping as deemed necessary.

### 2.4. Study Outcomes

The primary outcome of the study was the overall efficiency of LAT mapping, defined as the total number of electrograms acquired per map upon reaching the predefined filling threshold, comparing the LAT maps obtained with the Octaray and Pentaray catheters in either the left or right atrium. Several secondary outcomes were assessed, including mapping time, electrogram acquisition rate (i.e., the number of electrograms acquired per minute of mapping) and electrogram acquisition density (i.e., the number of electrograms acquired per square centimeter of the mapped atrial chamber). Moreover, the total number of electrograms acquired within possible scar areas of the left atrium (defined as regions with bipolar voltage < 0.5 mV) was compared between the Octaray and Pentaray catheters. The percentage of internal points (i.e., points located >7 mm from the atrial surface as identified by the electroanatomic mapping system) that do not contribute to propagation mapping, such as Coherent maps, was assessed for both catheters.

### 2.5. Statistical Analysis

The sample size calculation was based on a conservative estimate with a mean of 1000 electrograms per map, with a standard deviation of 300 electrograms, for the Pentaray catheter. Using a paired-sample *t*-test with a two-sided alpha of 0.05 and 90% power to detect an average difference of 250 electrograms with the Octaray catheter, a total of at least 18 patients with complete maps per atrium were determined to be required.

Categorical variables were presented as counts and percentages, and differences between groups were tested using the Chi-square test or Fisher’s exact test, depending on group sizes. Continuous variables were presented as medians with interquartile ranges because the assumption of normal distribution was frequently violated, as assessed by the Shapiro–Wilk test. Comparisons between groups were conducted using the Wilcoxon signed-rank test for paired data and the Wilcoxon rank-sum test for unpaired data. In all analyses a *p*-value of less than 0.05 was considered statistically significant. All analyses were conducted using the RStudio software package version 4.2.2.

## 3. Results

**Figure 2:**
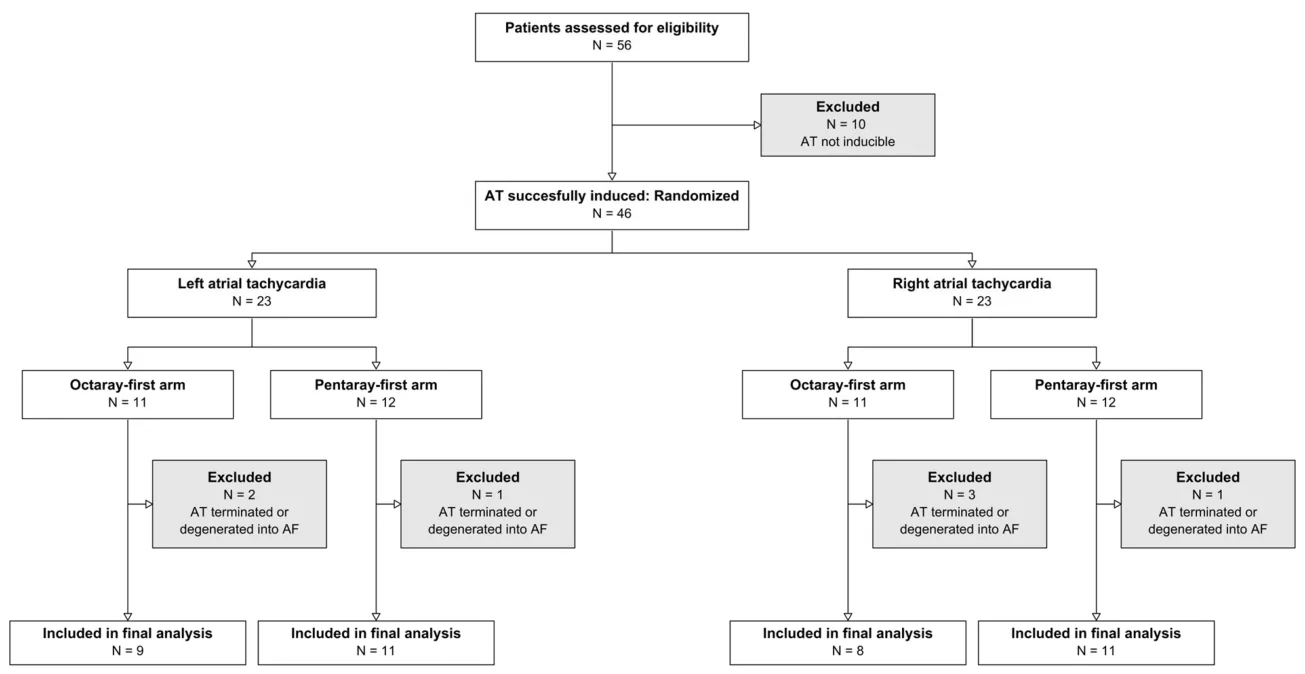
CONSORT flow diagram of patient enrollment, randomization, exclusion (grey shaded boxes) and final analysis.

A total of 56 patients (median age 65 years, 77% male) were screened (Figure 2). Of these, 46 patients were randomized to either an Octaray-first (n = 23) or Pentaray-first (n = 23) mapping strategy after successful AT induction. Subsequently, seven (15%) patients were excluded because AT terminated or degenerated into atrial fibrillation before completion of the mapping protocol and could not be successfully reinduced, leaving 39 patients for the final analysis.

Table 1 summarizes the baseline characteristics of the analyzed patients. Age, gender distribution, body mass index, prior ablation, prior cardiac surgery, and antiarrhythmic drug use were similar between both groups. No periprocedural major complications were observed up to 3 months of follow-up.

A total of 39 patients (20 with left AT and 19 with right AT) were analyzed to compare the performance of Octaray and Pentaray. All left ATs were classified as scar-related macro–reentrant ATs. In contrast, majority of right ATs were identified as typical cavotricuspid isthmus–dependent flutters (68%), while the remaining cases were either focal ATs (16%) or scar-related macro–reentrant ATs (16%). One left atrial AT was later determined to be a typical right atrial flutter, and one initially classified typical flutter was subsequently identified as a left-sided macro–reentrant AT. Both patients remained in the original groups to which they had been randomized. All patients were mapped with both catheters, as required by the cross-over design. Randomization only determined the order in which each catheter was used. As shown in Table 2, procedural parameters, including the primary endpoint (number of electrograms), were not influenced by the randomization group.

**Figure 3:**
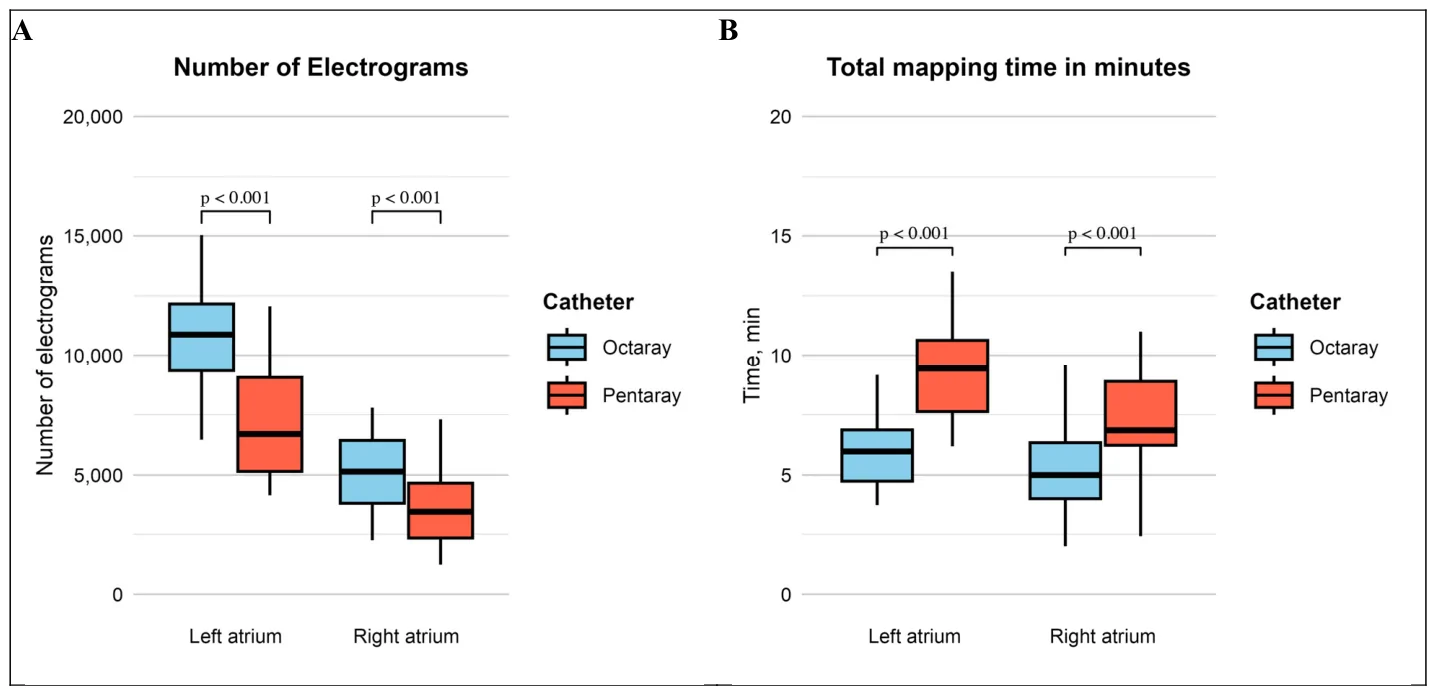
Number of electrograms (**A**) and mapping time (**B**) for the Pentaray and Octaray catheters, stratified by left and right atrium.

**Figure 4:**
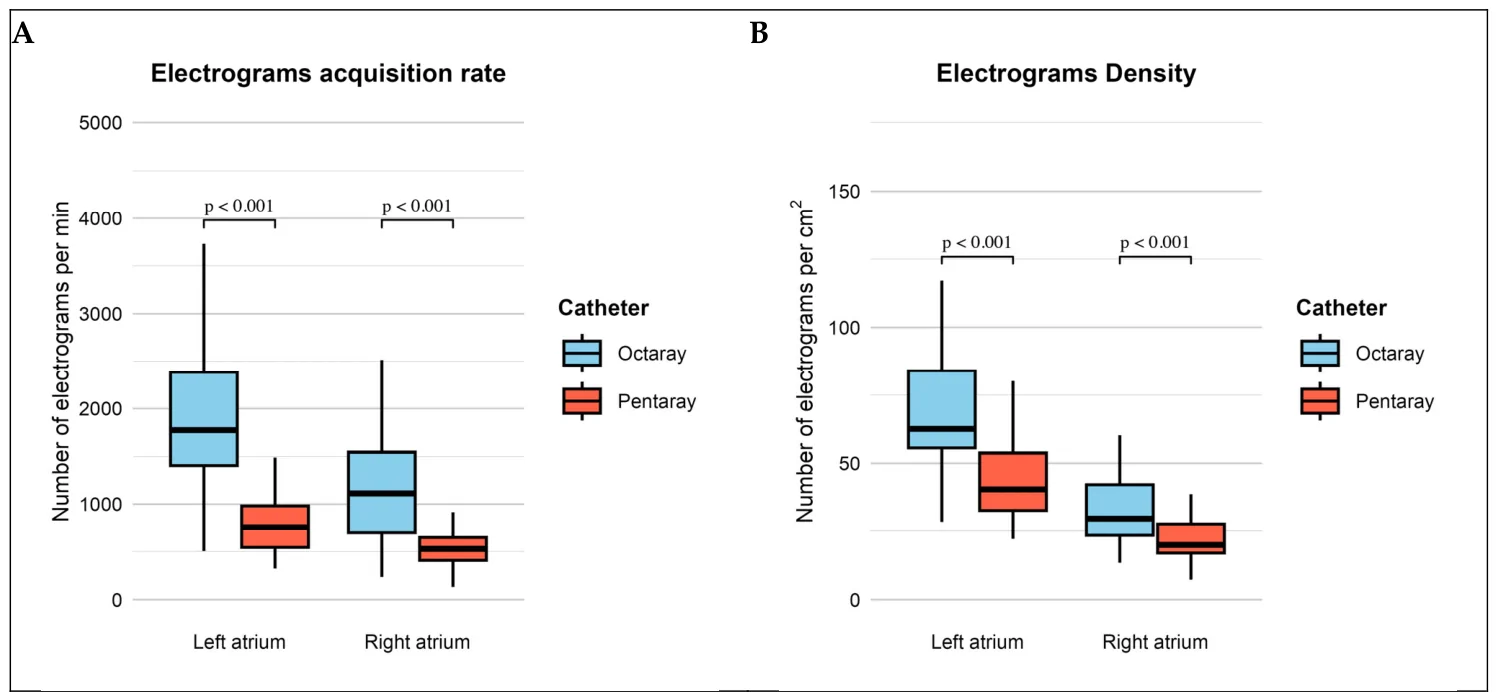
Electrogram acquisition rate (electrograms per minute; (**A**)) and electrogram density (electrograms per cm2; (**B**)) for the Pentaray and Octaray catheters, stratified by left and right atrium.

In the combined analysis of both left and right atria, the median number of electrograms per map was 7437 with the Octaray catheter compared with 4679 with the Pentaray (*p* < 0.001). The corresponding median mapping times were 5.4 min for Octaray and 8.1 min for Pentaray (*p* < 0.001). The acquisition rate was significantly higher with Octaray (median 1489 electrograms/min) than with Pentaray (median 602 electrograms/min; *p* < 0.001). Similarly, electrogram density was greater with Octaray compared with Pentaray (50.2 vs. 29.8 electrograms/cm^2^; *p* < 0.001). The number of electrograms and mapping times for the left and right atria are shown in Figure 3 and the corresponding electrogram acquisition rates and electrogram densities in Figure 4. The differences were more pronounced in the left atrium than in the right atrium.

**Figure 5:**
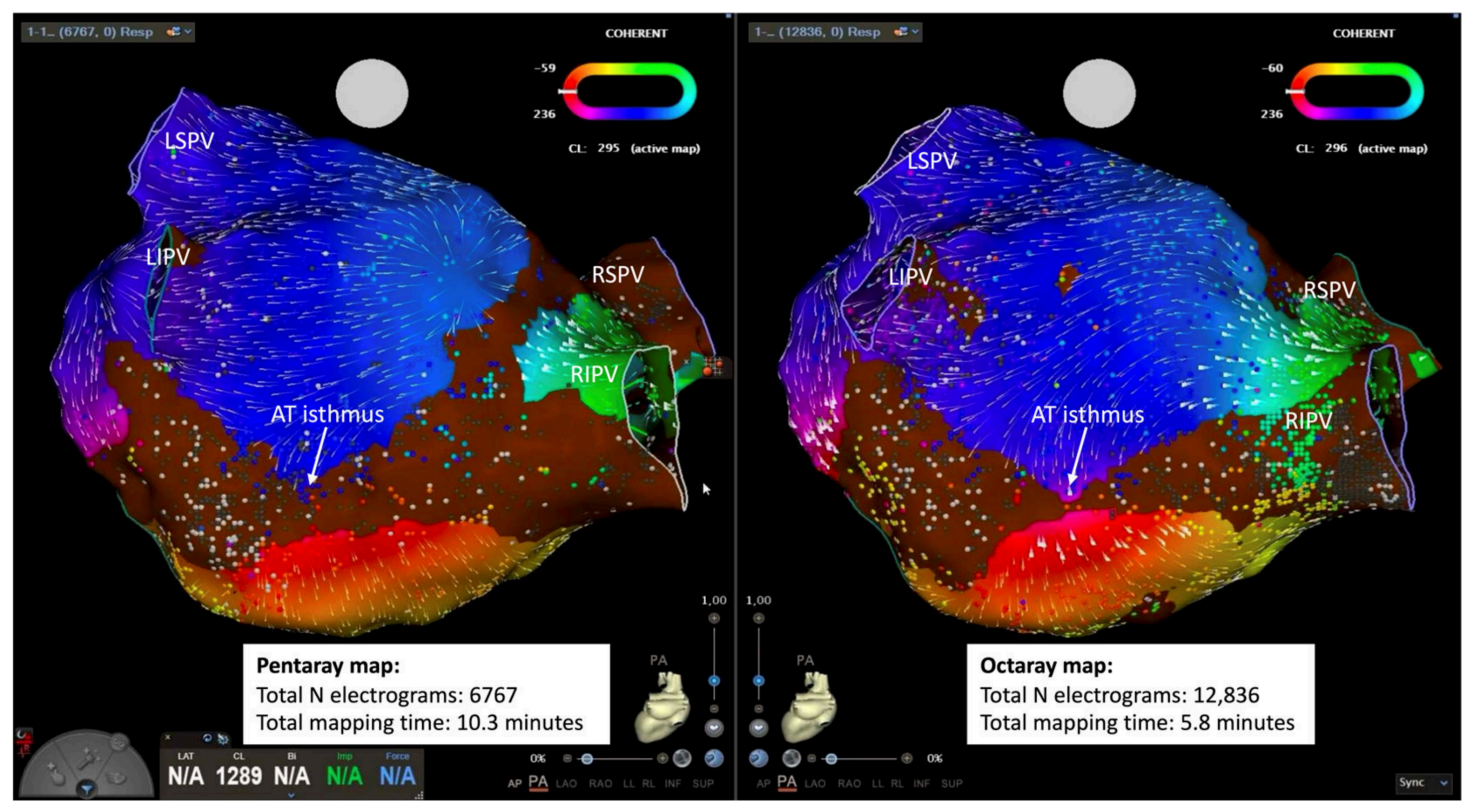
Representative Coherent activation maps acquired with the Pentaray catheter (**left**) and the Octaray catheter (**right**) in the same patient with scar-related left atrium AT. The higher electrogram density obtained with the Octaray catheter provides a more continuous and detailed representation of activation through the critical AT isthmus (arrow), enabling clearer visualization of slow conduction and facilitating interpretation of the propagation pattern. Total electrograms acquired and mapping times are shown for each catheter.

In a subset analysis restricted to left ATs, several sensitivity analyses were performed. The median percentage of low-voltage electrograms (i.e., bipolar voltage < 0.5 mV) was similar between Octaray and Pentaray maps (84% vs. 78%; *p* = 0.24). Within this subset of left ATs, the percentage of internal points was significantly lower with the Octaray (22%) than with the Pentaray catheter (40%; *p* = 0.021). This discrepancy was even more pronounced when only low-voltage signals (i.e., bipolar voltage < 0.5 mV) were considered (22% internal points with Octaray vs. 44% with Pentaray; *p* = 0.01). Using Coherent propagation mapping, the dense-scar regions (i.e., bipolar voltage < 0.03 mV) differed slightly between the two catheters, as shown in Figure 5 for a single illustrative case.

## 4. Discussion

In this randomized cross-over study, the Octaray catheter demonstrated significantly greater mapping efficiency than the conventional Pentaray catheter during mapping of regular atrial tachyarrhythmias. Octaray enabled acquisition of substantially more electrograms within shorter mapping times, resulting in a markedly higher acquisition rate and mapping density. These findings support the ability of Octaray to facilitate rapid, high-density electroanatomical mapping in routine clinical practice.

High-density mapping plays a central role in the characterization of complex AT mechanisms, particularly in patients with extensive atrial scar following prior ablation procedures [7]. Fractionated electrograms, anisotropic conduction, and heterogenous scar regions may complicate interpretation of activation patterns and prolong mapping procedures. A previous nonrandomized study in 10 patients with sustained AT demonstrated that the use of the Octaray catheter was associated with more electrograms per map (11,350 vs. 3628), a higher point density (59 vs. 18 electrograms/cm^2^), faster mapping speed (9 vs. 13 min) and higher electrogram acquisition rate (1332 vs. 308 electrogram/min) compared with the Pentaray catheter [16]. Using a cross-over design, the present randomized study confirms the improved mapping efficiency of the Octaray catheter compared with the Pentaray catheter. In our study, the electrogram acquisition rate and electrogram point density were approximately 2.5-fold and 1.7-fold higher, respectively, with the Octaray catheter. These advantages were more pronounced in left atrial scar-related ATs, which typically represent the most challenging substrate for activation mapping. While the current study was not designed to assess procedural success of long-term clinical outcomes, improved mapping efficiency may be particularly valuable in patients with unstable or short-lasting arrhythmias, where rapid map acquisition is essential.

Several technical characteristics may explain the observed differences between the two catheters. Octaray incorporates TRUEref™ technology to reduce far-field potentials [12,14]. The accuracy of the unipolar potentials is critical as the CARTO mapping system uses a wavefront annotation algorithm that automatically annotates the peak negative slopes (–dV/dT) of the unipolar electrograms. In addition, the Octaray catheter has 2.4-fold more electrodes, which enhances mapping speed, and also has smaller electrodes, improving the signal-to-noise ratio. Prior studies have demonstrated enhanced electrogram resolution and voltage map accuracy with Octaray compared with Pentaray [11,13,17]. The higher spatial voltage accuracy reduces the incidence of “false gaps” within prior ablation lines while also providing better identification of the critical isthmus (Figure 4). The present study extends these findings by demonstrating superior procedural mapping efficiency in a real-world clinical setting.

Although the proportion of low-voltage electrograms was similar between catheters, Octaray generated significantly fewer internal points, particularly within low-voltage regions. Internal points do not contribute meaningfully to propagation mapping. The lower proportion observed with Octaray therefore suggests improved catheter-tissue contact compared with Pentaray. This may be particularly relevant for algorithms such as Coherent Mapping, which rely on accurate spatial and temporal signal integration [10].

Several limitations should be acknowledged. The study was designed and powered to evaluate mapping efficiency rather than clinical outcomes. Accordingly, no conclusions can be drawn regarding procedural success, arrhythmia recurrence, or long-term outcomes. Similarly, given the cross-over design, the impact on procedural duration and other parameters such as the reduction in flouroscopy times cannot be assessed. The inclusion of typical flutters and focal right ATs, may have reduced the overall relative advantage of high-density mapping, which is more obvious in complex scar-related substrates. Finally, the study focused primarily on procedural and mapping metrics rather than detailed quantitative electrogram analysis.

In conclusion, the Octaray catheter enabled faster acquisition of high-density electroanatomical maps than the Pentaray catheter during mapping of regular ATs. These findings support the use of Octaray to improve procedural mapping efficiency during high-density mapping of complex ATs.

## Figures and Tables

**Table 1:** Baseline characteristics.

	Left Atrium			Right Atrium		
Characteristic	Octaray-First Arm N = 9	Pentaray-First Arm N = 11	*p*-Value	Octaray-First Arm N = 8	Pentaray-First Arm N = 11	*p*-Value
Age, years	69 (60–71)	68 (63–73)	0.8	69 (65–73)	62 (49–69)	0.2
Male sex	8 (89%)	8 (73%)	0.6	7 (88%)	8 (73%)	0.6
Body mass index, kg/m^2^	26 (22–27)	28 (23–32)	0.3	25 (24–27)	26 (24–31)	0.5
History of catheter ablation	7 (78%)	9 (82%)	>0.9	4 (50%)	2 (18%)	0.3
History of cardiac surgery	4 (44%)	6 (55%)	>0.9	4 (50%)	2 (18%)	0.3
Redo procedure	3 (33%)	4 (36%)	>0.9	2 (25%)	2 (18%)	>0.9
Class 3 antiarrhythmic drugs	2 (22%)	4 (36%)	0.6	3 (38%)	3 (27%)	>0.9

Data are presented as median (Q1–Q3) or n (%).

**Table 2:** Procedural parameters.

	Left Atrium			Right Atrium		
Characteristic	Octaray-First Arm N = 9	Pentaray-First Arm N = 11	*p*-Value	Octaray-First Arm N = 8	Pentaray-First Arm N = 11	*p*-Value
Number of Electrograms Pentaray	6722 (5333–8765)	6669 (4562–9878)	0.7	4311 (3089–4791)	2884 (1871–4655)	0.3
Time Pentaray, min	10.9 (6.6–13.5)	9.2 (8.0–10.2)	0.3	7.1 (6.6–8.6)	6.8 (5.2–10.2)	0.7
Number of Electrograms Octaray	10,872 (7449–11,048)	11,284 (9873–12,836)	0.3	6767 (4252–7458)	4852 (3803–5874)	0.11
Time Octaray, min	5.3 (4.7–6.3)	6.2 (5.0–7.0)	0.6	4.9 (3.8–5.6)	5.5 (3.9–7.0)	0.7
Procedural success	6 (67%)	11 (100%)	0.074	7 (88%)	10 (91%)	>0.9
Fluoroscopy time, min	19.0 (17.0–24.0)	14.6 (9.5–26.4)	0.4	7.6 (4.3–8.5)	8.4 (2.9–17.0)	>0.9

Data are presented as median (Q1–Q3) or n (%).

## Data Availability

The original contributions presented in this study are included in the article/Supplementary Material. Further inquiries can be directed to the corresponding author.

## Supplementary Materials

The following supporting information can be downloaded at https://www.mdpi.com/article/10.3390/jcdd13080379/s1, CONSORT 2025 checklist [18].
